# Stress degradation studies and stability-indicating TLC-densitometric method of glycyrrhetic acid

**DOI:** 10.1186/1752-153X-7-9

**Published:** 2013-01-17

**Authors:** Syed Ghulam Musharraf, Nayab Kanwal, Qamar ul Arfeen

**Affiliations:** 1 H.E.J. Research Institute of Chemistry, International Center for Chemical and Biological Sciences, University of Karachi, Karachi 75270, Pakistan

**Keywords:** Glycyrrhetic acid, Stress degradation, Electrospray ionization quadrupole time-of-flight mass spectrometry

## Abstract

**Background:**

Glycyrrhetic acid, a pentacyclic triterpenoid, possesses a broad range of pharmacological activities and serves as template to synthesize many bioactive drugs. This paper describes a simple, accurate, and sensitive stability-indicating TLC densitometric method for the determination of glycyrrhetic acid and its degradation product as per the ICH guidelines.

**Results:**

Separation was carried out on TLC aluminium sheet pre-coated with silica gel 60F254 using chloroform, methanol and formic acid (9:0.9:0.1, v/v). Compact spot for glycyrrhetic acid was found at *R*_f_ value of 0.42 ± 0.03. Densitometric analysis was carried out in the absorbance mode at *λ*_max_ 254 nm. Glycyrrhetic acid was found to be stable to the exposure of base, neutral, oxidation, dry heating treatment and wet heating treatment, but showed degradation under acidic and photochemical conditions. Moreover, fragmentation pattern of glycyrrhetic acid was developed by using a positive ion electrospray ionization quadrupole time-of-flight mass spectrometry (ESI-QqTOF-MS/MS) hybrid instrument. A photo-degraded product was characterized through comparison of mass spectrometric studies with glycyrrhetic acid.

**Conclusion:**

The developed stability-indicating TLC-densitometric method can be applied for routine analysis of glycyrrhetic acid in the presence of its degradation products.

## Background

*Glycyrrhiza glabra* Linn (Fabaceae) commonly known as Licorice, used worldwide as a natural sweetener and in certain cases, used as a flavor additive in the preparative of candies and foods. Moreover, powdered Licorice root is widely used in herbal drugs in the formulation of Ayurvedic and Chinese medicines. This herb has been reported with various biological activities including antitumour [1], anti-inflamatory [2], antiulcer [3], immunomodulatory [4], antimalarial [5], and anti-hypercholesterolmic [6]. The major constitute of Licorice is glycyrrhizin, which is potassium or calcium salt of glycyrrhizic acid. Glycyrrhizin is a pentacyclic triterpenoid glycoside, which is hydrolyzed to form aglycone glycyrrhetic acid [7]. Glycyrrhetic acid has shown antimicrobial [8] and anti-tumor activities [9]. Individual and simultaneously quantification of glycyrrhizin, 18*α*-glycyrrhetic acid, and 18*β*-glycyrrhetic acid in Licorice root and confectionary products have been developed with the help of various analytical tools including HPTLC [10-12], HPLC [13,14], microemulsion thin layer chromatography [15] and LC-ESI/MS/MS [16].

Majority of plants contain multiple compounds as active ingredients, which are frequently used in drugs. These active ingredients degraded and may alter their biological activities, therefore extensive study is required for estimation of their stability-indicating properties. The parent drug stability test guidelines (Q1AR) issued by International Conference on Harmonization (ICH) requires the stress testing to elucidate the inherent stability characteristics of the active substance. This guideline emphasizes the testing of those features which are susceptible to change during the storage under the influence of various environmental factors (temperature, light, humidity, oxidizing agent etc.). Quality, safety and efficacy must also be checked with validated stability-indicating testing methods [17,18].

A considerable attention is being paid to the development of stability-indicating TLC-densitometric method as it is fast, reliable and accurate technique. The major advantage of HPTLC over HPLC is that a number of samples can be processed at the same time by using a minute quantity of mobile phase, thus lowering the analysis time and cost per analysis. To the best of our knowledge, there is no report found for the stability-indicating method development of glycyrrhetic acid, while stability-indicating HPTLC method of glycyrrhizic acid has been reported [19]. In continuation of our work on the chromatographic method development of pharmaceutical important compounds [20-24], this paper describes a simple, accurate, and sensitive stability-indicating TLC densitometric method for the determination of glycyrrhetic acid (an active component of *Glycyrhiza glabra*) and its degradation product as per the ICH guidelines. Moerover, an extensive MS/MS study of glycyrrhetic acid and its photo-degraded products was also conducted using ESI-QqTOF-MS/MS and accurate mass measurements.

## Experimental

### Materials

Glycyrrhetic acid was purchased from Tokyo chemical Industry (TCI, Japan). Roots (S-1) and commercial extract (S-2) of *Glycyrrhiza glabra* were purchased from the local market. Precoated silica gel aluminum sheets (60F254, 20 cm × 20 cm) were perchased from Merck (Germany). Sodium hydroxide was purchased from BioM Laboratories (Cerritos, USA) while hydrochloric acid (HCl) and hydrogen peroxide (H_2_O_2_, 35% v/v) were obtained from Fisher Scientific (UK). Deionized water was obtained from Millipore Milli Q Plus System (Bedford, USA). All solvents used were of HPLC grade and purchased from Merck, (Germany).

## Instrumentation

### HPTLC analysis

Chromatography was performed by spotting the samples in the form of bands of width 6 mm with a CAMAG 100 μL syringe on pre-coated silica gel TLC aluminum sheets using Linomat V (CAMAG Muntenz, Switzerland) autosampler. A constant sample application rate of 0.1 μL/s was employed and the space between the two bands was 9.1 mm. 10 mL of mobile phase (chloroform: methanol: formic acid, 9:1:0.1, v/v) was used for linear ascending development and chromatogram was allowed to move to a distance of 80 mm, in twin trough glass chamber (CAMAG). The chamber saturation time for mobile phase was 10 minutes at 25 ± 2°C with relative humidity 42 ± 5%. The developed TLC plate was dried with the help of air dryer for 4 min. Video densitometry was carried out with CAMAG Reprostar III and scanning was performed on CAMAG TLC Scanner III at *λ*_max_ 254 nm which operates in reflection absorbance mode by winCATS software. Deuterium lamp with range between 190 nm and 400 nm was used as source. Evaluation was carried out *via* peak areas using linear regression.

### UPLC analysis

UPLC analysis were performed on Agilent 1200 Series, Rapid Resolution LC (RRLC) system, comprised of an Agilent 1260 binary pump with degasser, a high performance 1260 ALS autosampler with 1290 thermostat, a thermostated column compartment (1290 TCC), and a diode-array detector VL (1260 DAD VL). Data acquisition and integration was controlled by Agilent Technologies Chem-Station software. An Agilent Zorbax XDB-C18 column (50 × 3 mm i.d., 1.8 μm) was used. The mobile phase was a binary gradient system prepared from water (eluent A) and acetonitrile (eluent B), properly filtered and degassed for 15 min in ultra sonic bath before to use. The gradient program was: 50–60% B from 0–0.5 min, 60–65% B from 0.5–1 min, 65–70% B from 1–2 min, 70–75% B from 2–2.5 min, 75–100% B from 2.5–3 min, and 100–50% B from 3–4 min. The injection volume and flow rate were 5 μL and 1 mL min^-1^, respectively. The assay was performed at 40°C. The detection wavelength of DAD was 254 nm.

### ESI-QqTOF-MS/MS analysis

The standard and its photo-degraded products were dissolved in methanol and working dilution was prepared in 50:50 acetonitrile–water containing 0.1% tetrafluoro acetic acid (TFA). Analysis was performed by electrospray ionization (ESI) and collision-induced dissociation (CID), positive ion mode, on Qq-TOF-MS/MS instrument (QSTAR XL mass spectrometer Applied Biosystem/MDS Sciex, Darmstadt, Germany) coupled with 1100 HPLC system (Agilent). High-purity nitrogen gas was used as the curtain gas and collision gas delivered from Peak Scientific nitrogen generator. The ESI interface conditions were as follows: ion spray capillary voltage of 5500 V, curtain gas flow rate 20 L min^−1^, nebulizer gas flow rate 30 L min^−1^, DP1 60 V, DP2 10 V, and focusing potential of 265 V. The collision energy was swept from 20 to 45 eV for MS/MS analysis. Sample was introduced into the mass spectrometer using a Harvard syringe pump (Holliston, MA) at a flow rate of 5 μL min^−1^.

### Calibration curve of glycyrrhetic acid

Stock solution of glycyrrhetic acid (1 mg mL^-1^) was prepared in methanol. The stock solution was further diluted to obtain working standard of various concentrations including 200, 400, 600, 800, 1000, and 1200 ng spot^-1^ for calibration curve. Each working standard was spotted three times on TLC plate. The spotted plate was developed as mentioned in previous section. This practice was repeated six times to get an average standard calibration curve. The data of peak areas plotted against the corresponding concentrations were treated by linear least-square regression analysis.

### Method validation

Validation of the developed HPTLC method was carried out according to the ICH guidelines for sensitivity, precision, accuracy, robustness. The sensitivity of the method was determined with respect to LOD, LOQ, and correlation coefficient. Working solutions containing 200–1200 ng of glycyrrhetic acid were spotted on TLC aluminum sheet. In order to estimate limit of detection and limit of quantitation, calibration curve was used and were calculated by using following formula: LOD = 3.3 δ/S, LOQ = 10 δ /S where, δ = the residual standard deviation of regression line or the standard deviation of Y-intercept of regression line, S = the slope of the calibration curve. The LOD and LOQ were calculated as 3 and 10 times of the noise level, respectively. Furthermore, both were experimentally determined by diluting the known concentration of glycyrrhetic acid standard until the average responses were approximately three and ten times of the standard deviation of the responses for six replicate determinations. For method precision, the Intra- and inter-day variation for the determination of glycyrrhetic acid were carried out at three different concentration levels of 300, 500 and 700 ng spot^-1^. Repeated analyses were carried out in a same day for intra-day analysis while the same practice was repeated next day for inter-day analysis. Intra- and inter-day analyses were performed to check the repeatability and reproducibility of the method, respectively and results were statistically evaluated in terms of % R.S.D. In order to check the robustness, following parameters were intentionally changed within the range of ± 5% at three different concentration levels (300, 500 and 700 ng); mobile phase composition, time from spotting to chromatography, time from chromatography to scanning and chamber saturation time and using different type of TLC plates. Licorice root extract was prepared according to Cui Shufen *et al*. [15]. All samples were spotted on TLC plate and developed as mentioned in previous section. The accuracy of the method was assessed by performing recovery study at three different levels of glycyrrhetic acid (50%, 100%, and 150%).

### Preparation of forced degradation products

Stress degradation studies were performed using parallel synthesizer (Smart Start Synthesizer, Chem Speed Ltd., Switzerland) with sixteen reaction vessels. Stock solution containing 100 mg of glycyrrhetic acid in 100 mL of methanol was prepared. This stock solution (1 mg mL^-1^) was used for forced degradation studies in parallel synthesizer by refluxing the reaction mixtures for two hours at 80°C. After the reactions were completed, all the solutions were preserved at −20°C. Average peak areas of active components were analyzed after triplicate analysis.

For acidic hydrolysis, 3 mL of methanolic stock solution of glycyrrhetic acid (1 mg mL^-1^) were mixed with 3 mL of each 1N and 5N HCl separately and the resultant mixture solutions were refluxed for two hours at 80°C in the dark, in order to prohibit the possible degradative effects of light. 2 μL (1000 ng spot^-1^) of 1N and 5N HCl treated solutions of glycyrrhetic acid were applied on TLC sheet in triplicate and densitogram were developed. For alkaline hydrolysis, 3 mL of methanolic stock solution of glycyrrhetic acid (1 mg mL^-1^) were mixed with 3 mL of each 0.1N, 1N and 5N NaOH separately and the resultant mixture solutions were refluxed for two hours at 80°C in the dark. 2 μL (1000 ng spot^-1^) of 0.1N, 1N and 5N NaOH treated solutions of glycyrrhetic acid were applied on TLC sheet in triplicate and densitogram were developed. For neutral hydrolytic condition, 3 mL of methanolic stock solution of glycyrrhetic acid (1 mg mL^-1^) were mixed with 3 mL of milli Q water and the resultant solution was refluxed for two hours at 80°C in the dark. 2 μL (1000 ng spot^-1^) of treated solution of glycyrrhetic acid was applied on TLC sheet in triplicate and densitogram were developed. For wet heating, 3 mL of methanolic stock solution of glycyrhhetic acid was refluxed for two hours at 80°C in the dark. 1 μL (1000 ng spot^-1^) of resultant solutions were applied on TLC sheet in triplicate and densitogram were developed. Oxidative degradation was carried out by taken 3 mL stock solutions of glycyrrhetic acid mixed with 3 mL of H_2_O_2_ (35% v/v) and the resultant solutions were refluxed for two hours at 80°C in the dark and 2 μL (1000 ng spot^-1^) of resultant solutions were applied on TLC sheet in triplicate and densitogram were developed.

Photo-degradation studies were carried out by the exposure of stock solution of glycyrrhetic acid to direct sunlight for three days from 8 to 18 hrs at 30 ± 2°C. 1 μL (1000 ng spot^-1^) of resultant solutions were applied on TLC sheet in triplicate and densitogram were developed. Dry heating was performed by keeping standard glycyrrhetic acid in oven at 90°C for 4 hrs. 1 mg of treated standard was dissolved in 2 mL of methanol and 2 μL (1000 ng spot^-1^) of resultant solution of glycyrrhetic acid was applied on TLC plate in triplicate and densitogram were developed. For oxidation reaction at room temperature, 3 mL stock solution of glycyrrhetic acid was mixed with 3 mL of H_2_O_2_ (35% v/v) and the resultant solutions was kept for 24 hours at room temperature. 2 μL (1000 ng spot^-1^) of resultant solutions were applied on TLC sheet in triplicate and densitogram were developed.

## Results and discussion

### Method optimization

**Figure 1:**
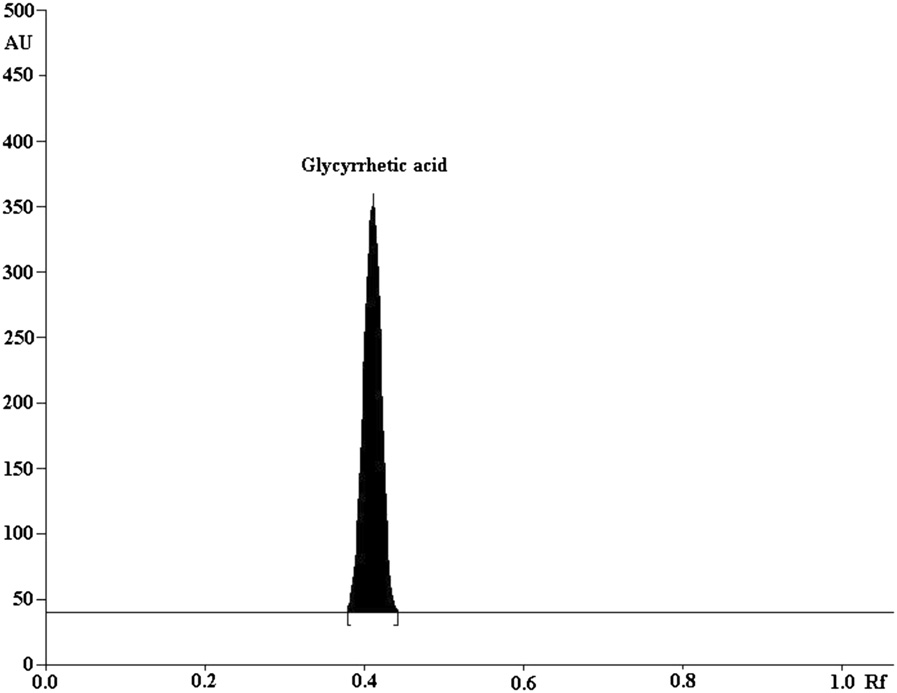
**HPTLC Chromatogram of standard glycyrrhetic acid (1000 ng spot**^**-1**^**): peak 1 (*R***_**f **_**= 0.42 ± 0.03), mobile phase; chloroform: methanol: formic acid (9:0.9:0.1).**

The TLC procedure was optimized with a view to develop stability-indicating assay method. Both the standard and degraded products were spotted on the TLC plates and developed in different solvent systems. Different mobile phases were tried to resolve glycyrrhetic acid from its degraded product. Different compositions of mobile phase and resulting *R*_f_ values of standards are summarized in (see Additional file 1: Table S1). Suitable separation with best resolution was achieved with chloroform: methanol: formic acid in the ratio of (9:0.9:0.1, v/v) which showed sharp and symmetrical peaks with *R*_f_ value of glycyrrhetic acid at 0.42 ± 0.03 (Figure 1). Well defined spots were obtained when the chamber was saturated with the mobile phase for 10 min at room temperature. The linear regression data for the calibration curve (n = 6) showed a good linear relationship with *r*^2^ ± SD = 0.998 ± 0.0019 over concentration range of 200–1200 ng spot^-1^ with respect to the peak area. Calibration curve for glycyrrhetic acid is shown in (Additional file 1: Figure S1).

### Method validation

Repeatability and reproducibility of the method was determined using intra and inter-day analysis. The % relative standard deviation (R.S.D. %) for glycyrrhetic acid were found to be less than 1% which shows good precision of proposed method (Table 1). Limit of detection at 3:1 signal to noise ratio was found to be 1.56 ng spot^-1^ while limit of quantification at 10:1 signal to noise ratio was found to be 4.74 ng spot^-1^, which indicate the adequate sensitivity of the method. For robustness analysis, the standard deviation of standard levels (300, 500 and 700 ng) was estimated for each parameter. Mean % R.S.D. was 0.94 for varying in mobile phase composition, 0.79 for varying chamber saturation time, 1.73 for the use of different TLC plates, 2.41 for varying time from spotting to chromatography, 1.25 for varying time from chromatography to scanning (Table 2). Spiking studies showed recovery of glycyrrhetic acid (96.7- 101.5%) in Licorice root extract ([see Additional file 1: Table S2]). The linear regression data and the method validation results are summarized in Table 3.

**Table 1:** Precision and accuracy for quality control standard of glycyrrhetic acid

**Analyst 1**							**Analyst 2**		
	**Intra-day**			**Inter-day**					
**Conc.**	Found^*^	**R.S.D.**	**Accuracy**	Found^*^	**R.S.D.**	**Accuracy**	Found^*^	**R.S.D.**	**Accuracy**
(ng)	(ng)	%	(%)	(ng)	%	(%)	(ng)	%	(%)
300	303.609±1.21	0.399	101.203	303.868±1.88	0.621	101.289	302.073±1.97	0.650	100.691
500	504.566±1.01	0.200	100.913	511.555±1.62	0.317	102.311	508.012±2.26	0.445	101.602
700	708.767±1.15	0.162	101.252	709.6±1.83	0.258	101.371	708.812±1.73	0.244	101.259

^*^Values are mean of n = 9.

**Table 2:** Robustness Study (n = 3)

**Standard level**	**Mobile phase composition**		**Saturation time**		**Nature of TLC**		**Time from spotting to chromatography**		**Time from chromatography to scanning**	
**Amount (ng/spot)**	**Amount detected (ng ± S.D.)**	**R.S.D. (%)**	**Amount detected (ng ± S.D.)**	**R.S.D. (%)**	**Amount detected (ng ± S.D.)**	**R.S.D. (%)**	**Amount detected (ng ± S.D.)**	**R.S.D. (%)**	**Amount detected (ng ± S.D.)**	**R.S.D. (%)**
300	306.16 ± 3.78	1.22	304.45 ± 3.66	1.20	`302.81 ± 2.72	0.89	303.39 ± 2.86	0.94	301.7 ± 5.67	1.90
500	512.97 ± 4.15	0.81	514.78 ± 3.32	0.64	515.1 ± 5.06	0.98	511.88 ± 4.01	0.78	517.9 ± 5.46	1.05
700	716.63 ± 5.62	0.78	711.2 ± 3.82	0.54	717.35 ± 10.24	1.42	720.48 ± 4.99	0.69	715.58 ± 5.76	0.806

**Table 3:** Summary of validation parameters

**Parameter**	**Data of standard Glycyrrhetic acid (at *λ***_**max **_**254nm)**
Linearity range	200-1200 ng spot^-1^
Correlation coefficient *r*^2^ ± SD	0.998 ± 0.0019
Limit of detection (LOD)	1.56 ng spot^-1^
Limit of quantitation (LOQ)	4.74 ng spot^-1^
Y = mx + c	5.736x + 664.3
Slope ± SD	5.76 ± 0.58
Intercept ± SD	664.3 ± 25.54
Intra-day analysis (n = 3), % RSD	0.253
Inter-day analysis (n = 3), % RSD	0.3986
Robustness	Robust
Specificity	Specific

### Stability-indicating property

**Figure 2:**
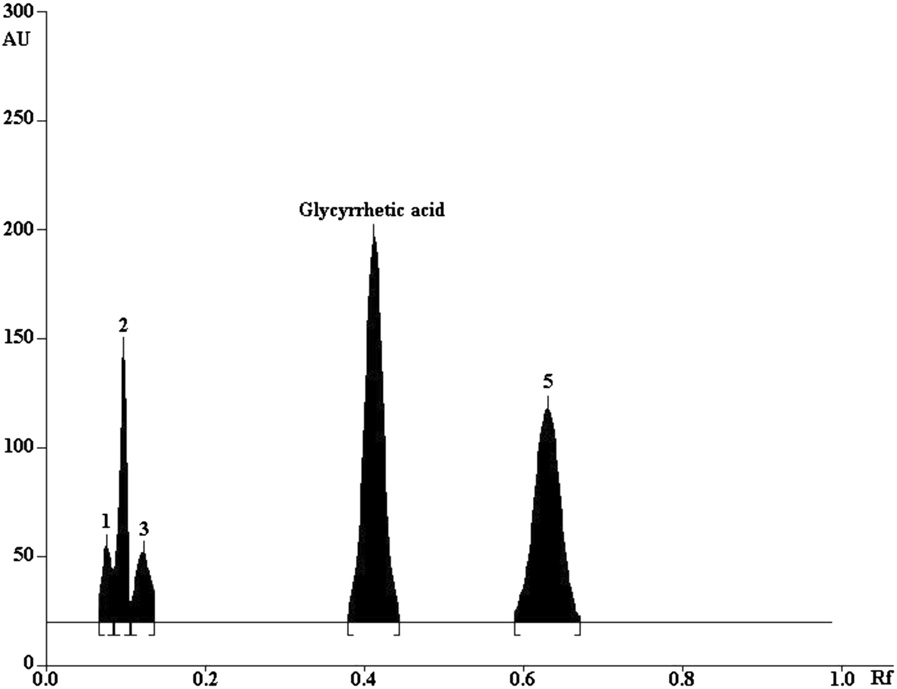
**HPTLC Chromatogram of acid (5N HCl, reflux for 2h at 80°C) treated glycyrrhetic acid: peak 1, degradant (*R***_**f **_**= 0.08); peak 2, degradant (R**_**f **_**= 0.1); peak 3, degradant (*R***_**f **_**= 0.12); peak 4, glycyrrhetic acid (*R***_**f **_**= 0.42); peak 5, degradant (*R***_**f **_**= 0.64).**

The chromatogram of glycyrrhetic acid treated with acid and sunlight showed well-separated peak of glycyrrhetic acid as well as some additional peaks (Figure 2, 3A). No degradation was observed after exposing the glycyrrhetic acid to stressed conditions of base, neutral, hydrogen peroxide, wet heating, and dry heating. Results of the stress degradation studies are summerized in Table 4. Glycyrrhetic acid showed 63.7% and 74.6% degradation under 1N HCl and 5N HCl conditions, respectively. Degraded peaks were observed at *R*_f_ 0.08, 0.1 and 0.64 (major), while in strong acidic medium (5N HCl) an additional peak generated at *R*_f_ 0.12. Peak observed at *R*_f_ 0.64 was the artifact of glycyrrhetic acid that is glycyrrhetic acid methyl ester, which was confirmed by HR-EI-MS and ^1^H NMR. Under photochemical conditions, glycyrrhetic acid showed 33.7% degradation, while the degraded products were observed at *R*_f_ 0.35, 0.38, 0.49. Under basic hydrolysis, salt was formed. UPLC chromatogram of glycyrrhetic acid treated with acid, base, and neutral are shown in (see Additional file 1: Figure S2). Moreover, screening of Licorice root extract (S-1) and its commercially available extract (S-2) showed the presences of its phtoto-degraded product at *R*_f =_ 0.49 (Figure 4).

**Figure 3:**
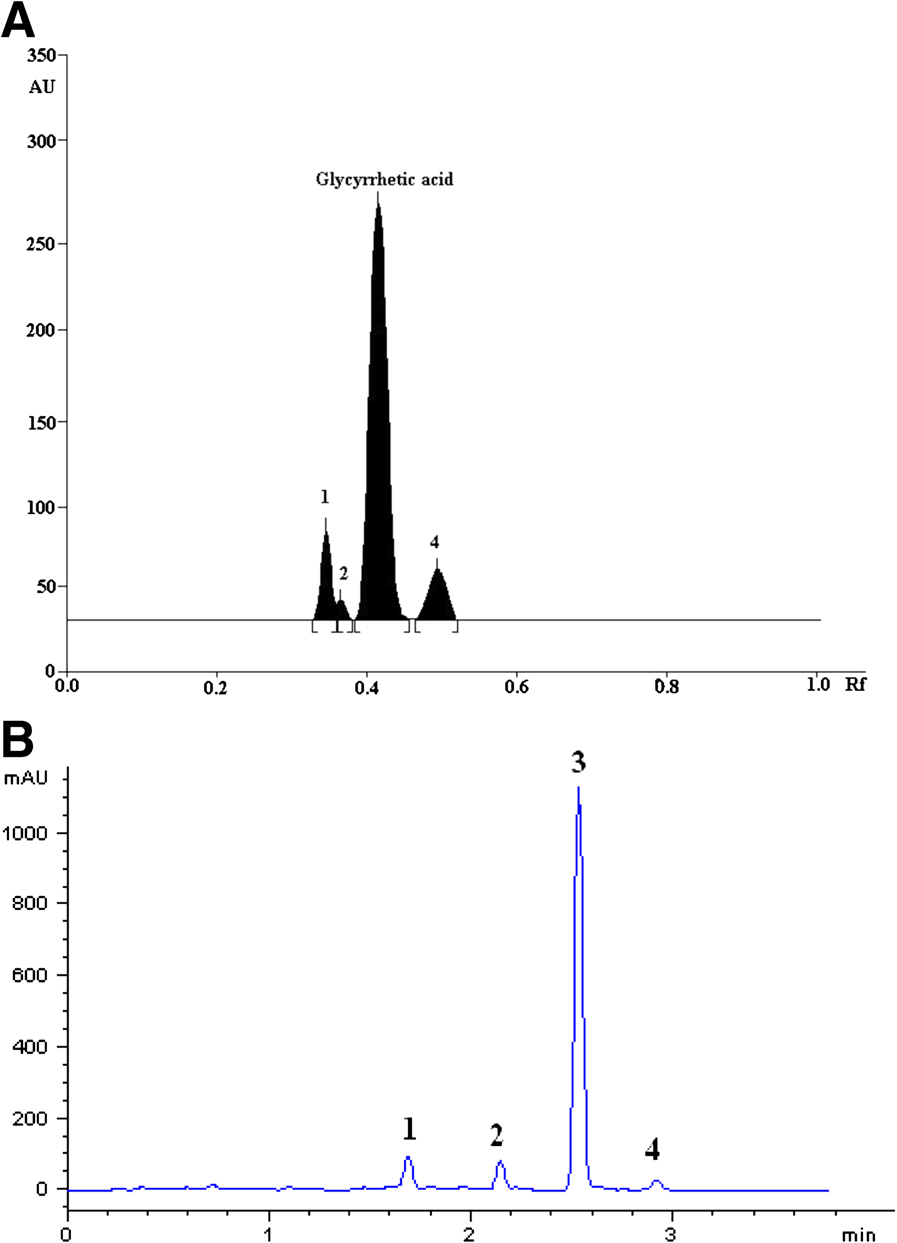
**HPTLC Chromatogram (A) and UPLC chromatogram (B) of photo-degraded standard glycyrrhetic acid kept in sunlight for 24hr (1000 ng/spot): peak 1, degradant (*R***_**f **_**= 0.35 and R**_**t **_**= 1.687); peak 2, degradant (*R***_**f **_**= 0.38 and R**_**t **_**= 2.142); peak 3, glycyrrhetic acid (*R***_**f **_**= 0.42 and R**_**t **_**= 2.531); peak 4, degradant ( *R***_**f **_**= 0.49 and R**_**t **_**= 2.916).**

**Table 4:** Summary of stress degradation studies of glycyrrhetic acid

**Degradation conditions**	**Time (hour)**	***R***_**f **_**of degraded products**	**Compound remained (ng /1000ng±S.D., n=3)**	**% Recovery**
**Acidic hydrolysis **^a^				
1N HCl	2	0.08, 0.1,0.64	362.19±7.7	36.21
5N HCl	2	0.08,0.1, 0.12, 0.64	253.83±1.5	25.38
**Basic hydrolysis **^a^				
0.1N NaOH	2	Not detected	999.9±5.2	99.9
1N NaOH	2	Not detected	892.9±11	89.2
5N NaOH	2	Not detected	-	100^b^
**Oxidation**				
35%v/v H_2_O_2_^a^	2	Not detected	993.07±5.5	99.3
Oxidation at room temp	24	Not detected	987.8±6.4	98.7
**Neutral hydrolysis **^a^	2	Not detected	997.09±7.7	99.7
**Wet heating **^a^	2	Not detected	999.47±9.5	99.9
**Dry heating**	4	Not detected	999.58±6.2	99.9
**Photostability- daylight**	24	0.35,0.38,0.49	663.74±5.1	66.3

^a^Reflux in parallel synthesizer at 80°C.

^b^Recover in salt form.

**Figure 4:**
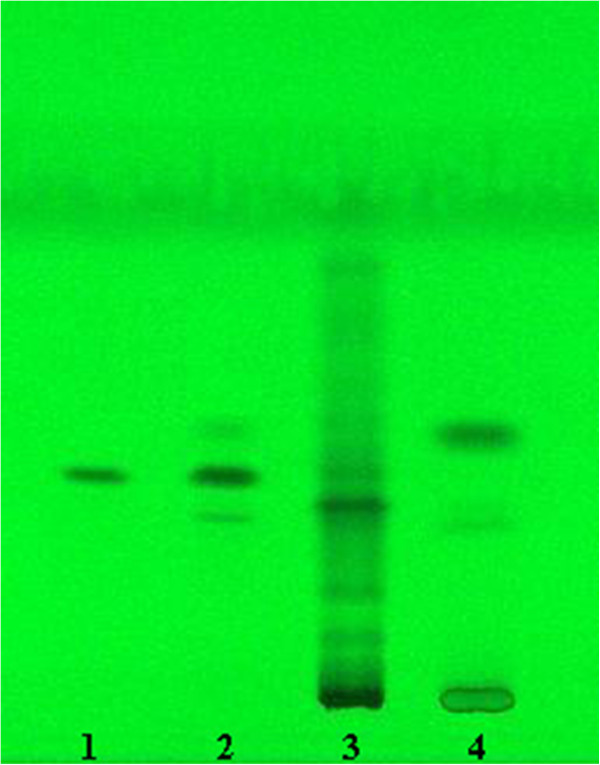
**Video densitogram showing spots of (1) standard glycyrrhetic acid, (2) photo-degradation mixture of glycyrrhetic acid, (3) MeOH extract of *Glycyrrhiza glabra *root (S-1), (4) commercial extract of *Glycyrrhiza glabra *(S-2).**

### MS/MS analysis of glycyrrhetic acid and its photo-degradation products

UPLC analysis of photo-degradation reaction mixture of glycyrrhetic acid showed the elution of degraded products within 3 min (Figure 3B). The retention times (R_t_) and proposed formula of all the peaks are shown in Table 5. ESI-QqTOF-MS (positive mode) scan of glycyrrhetic acid and its degradation products showed peak [M+H]^+^ at *m/z* 471.3488, 485.3263, 487.3450, and 457.3636, corresponding to the molecular formulae C_30_H_47_O_4_ (calc. 471.3468), C_30_H_45_O_5_ (calc. 485.3263), C_30_H_47_O_5_ (calc. 487.3423) and C_30_H_49_O_3_ (calc. 457.3676), respectively.

**Table 5:** LC-ESI-MS/MS analysis of photo-degradation mixture of glycyrrhetic acid

**Peak**	**Rt (min)**	**Proposed formula**	**Observed mass**	**Calculated mass**	**Error (ppm)**	**Characteristic MS/MS fragment ions**
Peak 1	1.687	C_30_H_45_O_5_	485.3263	485.3261	0.3601	467, 449, 431, 421,403, 385, 259, 233, 187
Peak 2	2.142	C_30_H_47_O_5_	487.3450	487.3423	5.4374	451,423,405,387, 317, 271,235,189,175
Glycyrrhetic acid ( Peak 3)	2.531	C_30_H_47_O_4_	471.3488	471.3468	4.0588	453, 425, 407, 389, 317, 271, 263, 235, 217, 189, 149
Peak 4	2.916	C_30_H_49_O_3_	457.3636	457.3676	−8.7945	439, 411, 393, 343, 249, 203, 191

**Figure 5:**
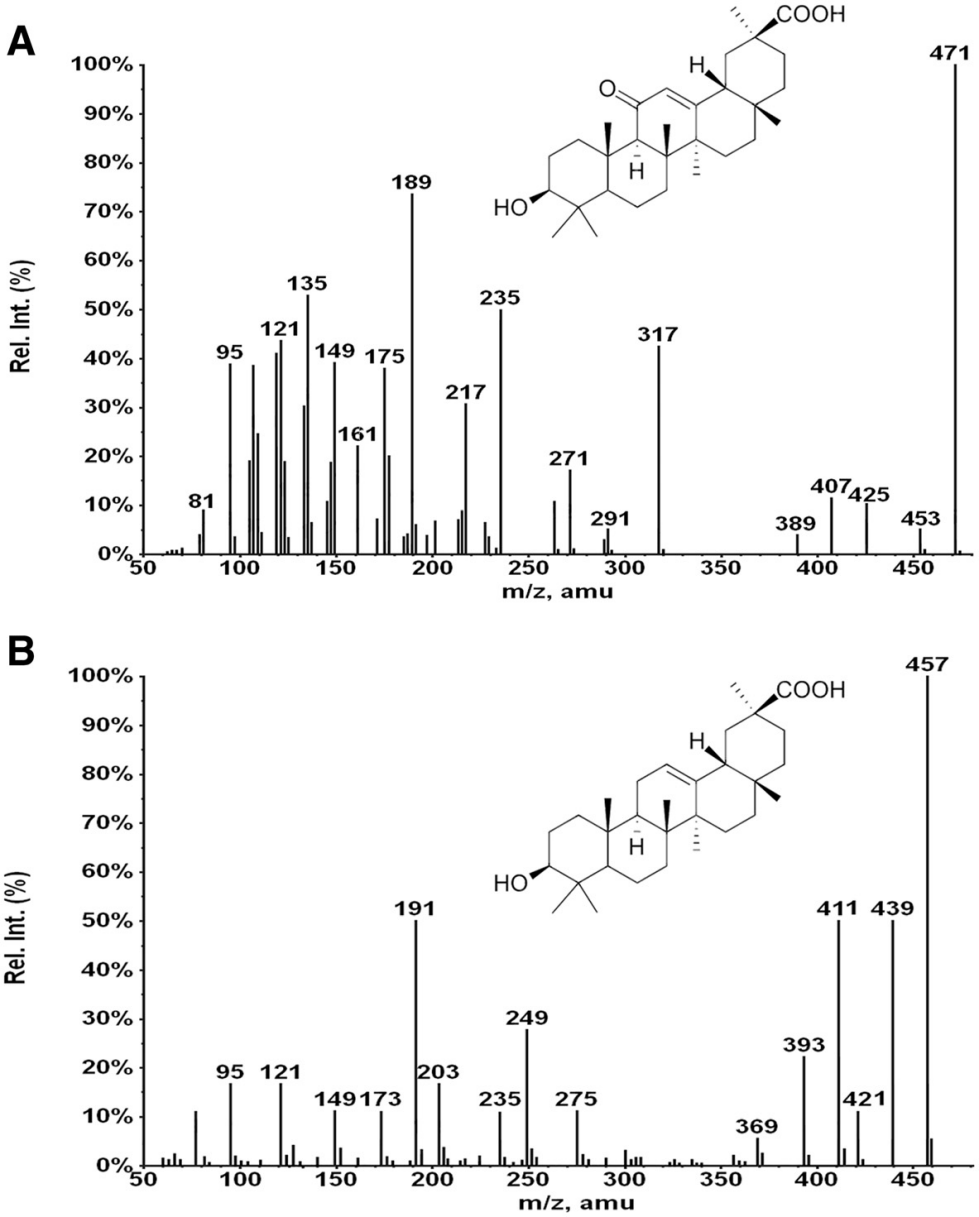
CID-MS/MS spectra of (a) glycyrrhetic acid and (b) its photo-degradation products, peak 4.

**Scheme 1:**
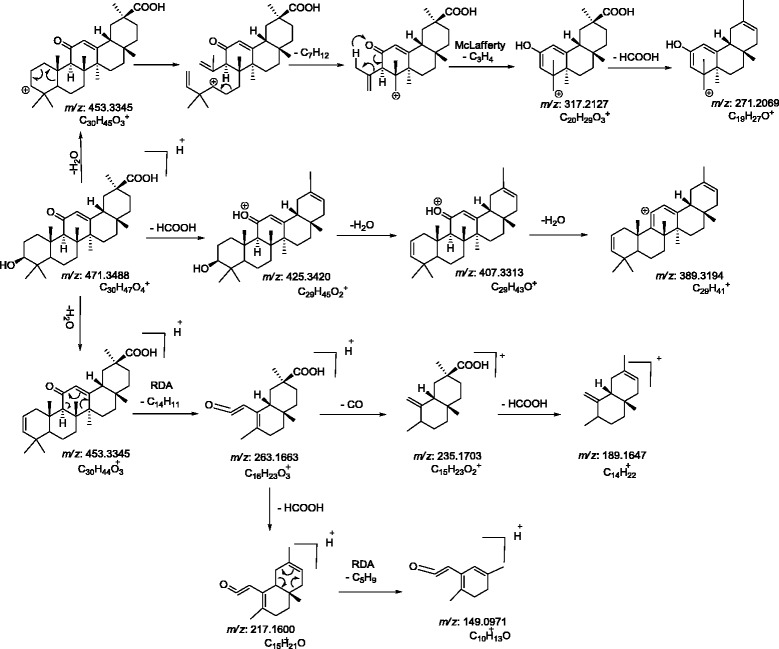
Proposed CID-MS/MS fragmentation pathway of glycyrrhetic acid.

The ESI-MS/MS spectrum of [M+H]^+^ ions (*m/z* 471) of glycyrrhetic acid (Peak 3), showed neutral loses of H_2_O and HCOOH from [M+H]^+^ at *m/z* 453 [M+H - H_2_O]^+^, *m/z* 425 [M+H - HCOOH]^+^, *m/z* 407 [M+H - HCOOH - H_2_O]^+^, *m/z* 389 [M+H- HCOOH- 2H_2_O]^+^. Fragment at *m/z* 263 was supposed to be generated from *m/z* 453 by retro Diel-Alder cleavage of ring C, and fragment at *m/z* 235 was observed by the loss of CO from *m/z* 263. Subsequent neutral loss of formic acid from *m/z* 235 formed the product ion at *m/z* 189. Fragment at *m/z* 317 was supposed to be formed from *m/z* 453 by the loss of C_10_H_16_ moiety. Subsequent neutral loss of formic acid from *m/z* 317 formed the product ion at *m/z* 271. The MS/MS spectra and mechanistic fragmentation pathway of glycyrrhetic acid are shown in Figure 5a and Scheme 1, respectively.

The ESI-MS/MS spectrum of [M+H]^+^ ions of *m/z* 485 (Peak 1) and *m/z* 487 (Peak 2) , showed only 14 and 16 amu increase from glycyrrhetic acid, respectively. Characteristic MS/MS fragments are summarized in Table 5. These compounds were not isolated due to further degradation during chromatographic processing, and their structures were not characterized due to miss matched fragmentation pattern with the available structure according to the molecular formula in Dictionary of Natural Products.

**Scheme 2:**
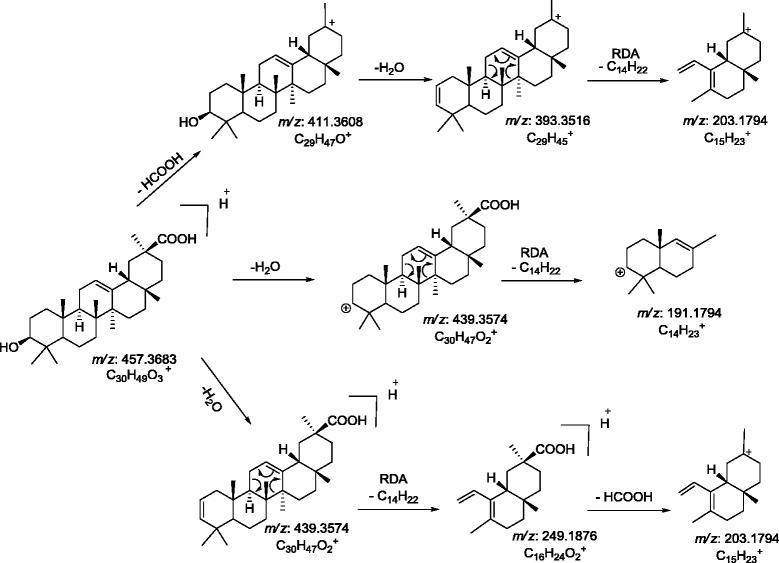
Proposed CID-MS/MS fragmentation pathway of photo-degradation product of glycyrrhetic acid (Peak 4).

The ESI-MS/MS spectrum of [M+H]^+^ ions (*m/z* 457) of peak 4, eluting at retention time (R_t_) 2.916 in UPLC, showed neutral loss of H_2_O and HCOOH from [M+H]^+^ generated ions at *m/z* 439 [M+H - H_2_O]^+^, *m/z* 411 [M+H - HCOOH]^+^, *m/z* 393 [M+H – HCOOH - H_2_O]^+^ respectively. Fragment due to retro Diel-Alder cleavage of ring C was observed at *m/z* 249, *m/z* 203, and *m/z* 191 from fragment *m/z* 439, *m/z* 393, *and m/z* 439, respectively. The MS/MS spectra and mechanistic fragmentation pathway are shown in Figure 5b and Scheme 2, respectively. Degradant (peak 4) showed only 14 amu difference in comparison to glycyrrhetic acid, while MS/MS spectra showed similar fragmentation pattern as observed in glycyrrhetic acid. The structure of peak 4 was proposed as 11-deoxy-glycyrrhetic acid. Isolation of this degraded product was carried out and the structure was also confirmed by ^1^H and ^13^C NMR techniques.

All proposed fragmentation pathways have been confirmed by accurate mass measurements, which are summarized in (Additional file 1: Table S3). Degraded product was identified through comparative MS/MS studies with glycyrrhetic acid with the help of data base (Dictionary of Natural products). Degradation product was searched in the update Dictionary of Natural Products (DNP, version 20.2) on the basis of deprotonated molecular mass and respective formulae for the identification of compound. In the case of more than one match, the search was narrowed down to the plant species (*Glycyrrhiza glabra*) and to the other species of *Glycyrrhiza*.

## Conclusion

The developed and validated TLC-densitometric method is precise, accurate, and stability-indicating for the quantification of glycyrrhetic acid in the presences of its degradation products. Glycyrrhetic acid showed extensive degradation in acidic and photochemical stress conditions, while stable to alkaline, neutral, oxidative, dry heating and wet heating stress conditions. A photo-degradation product was also characterized with the help of the ESI-QqTOF-MS/MS experiments combined with accurate mass measurements of precursor and fragment ions. The results showed the importance of appropriate light protection during the drug development process, storage and handling.

## Competing interests

Authors declare that they have no competing interests.

## Authors’ contributions

SGM: Participated in the experimental designing and method optimization. NK: Performed the experiments and wrote the manuscript. QA: Involved in the useful discussion and also participated in experimental work. All authors read and approved the final manuscript.

## Supplementary Material

Additional file 1: Figure S1 Calibration curve for glycyrrhetic acid (200-1200 ng spot^-1^) at 254 nm. **Figure S2.** UPLC Chromatogram of (A) acidic hydrolysis: peak 1, degradant (R_t_ = 0.21); peak 2, degradant (R_t_ = 0.278); peak 3, degradant (R_t_ = 0.441); peak 4, glycyrrhetic acid (R_t_ = 2.531); peak 5, degradant (R_t_ = 3.776), (B) basic hydrolysis (C) neutral hydrolysis. **Table S1.** R_f_ values of glycyrrhetic acid in different mobile phases. **Table S2.** Recovery studies of glycyrrhetic acid (n=3). **Table S3.** Elemental composition of daughter ions of glycyrrhetic acid (*m/z* 471), and Peak 4 (*m/z* 457). Click here for file
