# Supplementary material for: Stress degradation studies and stability-indicating TLC-densitometric method of glycyrrhetic acid
Source: Chem Cent J. 2013 Jan 17;7:9. doi: 10.1186/1752-153X-7-9 (PMC3637560; doi:10.1186/1752-153X-7-9)
Supplement: Additional file 1: Figure S1 — Calibration curve for glycyrrhetic acid (200-1200 ng spot-1) at 254 nm. Figure S2. UPLC Chromatogram of (A) acidic hydrolysis: peak 1, degradant (Rt = 0.21); peak 2, degradant (Rt = 0.278); peak 3, degradant (Rt = 0.441); peak 4, glycyrrhetic acid (Rt = 2.531); peak 5, degradant (Rt = 3.776), (B) basic hydrolysis (C) neutral hydrolysis. Table S1. Rf values of glycyrrhetic acid in different mobile phases. Table S2. Recovery studies of glycyrrhetic acid (n=3). Table S3. Elemental composition of daughter ions of glycyrrhetic acid (m/z 471), and Peak 4 (m/z 457). [file 1752-153X-7-9-S1.docx]

**Additional file1**

**Figure S1** Calibration curve for glycyrrhetic acid (200-1200 ng spot^-1^) at 254 nm.

**Figure S2** UPLC Chromatogram of (A) acidic hydrolysis: peak 1, degradant (R_t_ = 0.21); peak 2, degradant (R_t_ = 0.278); peak 3, degradant (R_t_ = 0.441); peak 4, glycyrrhetic acid (R_t_ = 2.531); peak 5, degradant (R_t_ = 3.776), (B) basic hydrolysis (C) neutral hydrolysis.

**Table S1** R_f_ values of glycyrrhetic acid in different mobile phases.

**Table S2** Recovery studies of glycyrrhetic acid (n=3)

**Table S3** Elemental composition of daughter ions of glycyrrhetic acid (*m/z* 471), and Peak 4 (*m/z* 457).


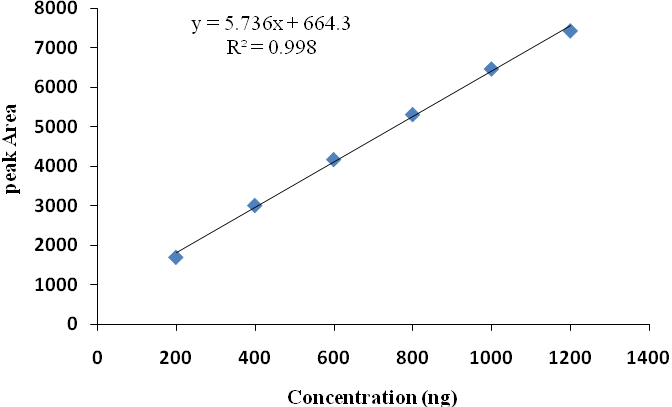


**Figure S1** Calibration curve for glycyrrhetic acid (200-1200 ng spot^-1^) at 254 nm.


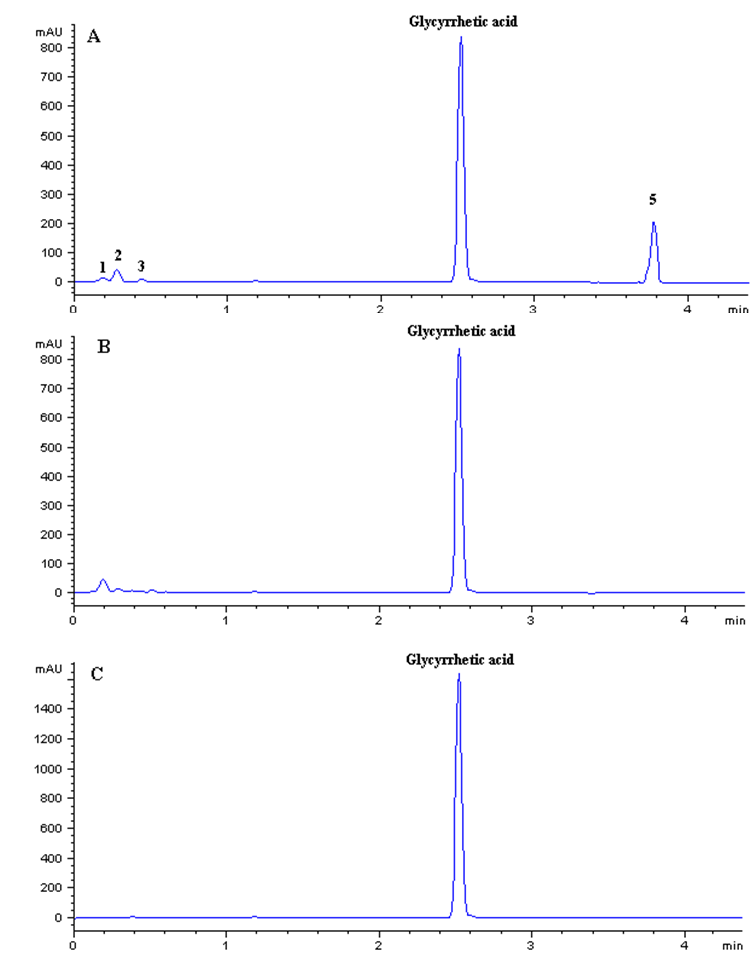


**Figure S2** UPLC Chromatogram of (A) acidic hydrolysis: peak 1, degradant (R_t_ = 0.21); peak 2, degradant (R_t_ = 0.278); peak 3, degradant (R_t_ = 0.441); peak 4, glycyrrhetic acid (R_t_ = 2.531); peak 5, degradant (R_t_ = 3.776), (B) basic hydrolysis (C) neutral hydrolysis.

**Table S1 R_f_ values of glycyrrhetic acid in different mobile phases**

| S.No. | Compositions (v/v) | Proportions | *R*_f_ |
| --- | --- | --- | --- |
| 1 | Toluene-ethyl acetate-formic acid | 8:2:0.12 | 0.24 |
| 2 | Chloroform-methanol-formic acid | 9:0.9:0.1 | 0.42 |
| 3 | Dichloromethane-methanol | 9.4:0.6 | 0.70 |
| 4 | Hexane: acetone-acetic acid | 4:6:0.2 | 0.82 |
| 5 | Chloroform-methanol | 9:1 | 0.83 |

**Table S2 Recovery studies of glycyrrhetic acid (n=3)**

| Standard spiked % | Amount of glycyrrhetic acid present (ng spot^-1^) | Theoretical content (ng spot^-1^) | Average experimental  contents (ng spot^-1^) | % Recovery |
| --- | --- | --- | --- | --- |
| 50 | 108.48 | 162.72 | 157.36 | 96.71 |
| 100 | 108.48 | 216.96 | 211.24 | 97.36 |
| 150 | 108.48 | 271.2 | 275.52 | 101.59 |

**Table S3 Elemental composition of daughter ions of glycyrrhetic acid (*m/z* 471), and Peak 4 (*m/z* 457).**

|  | Proposed formula | Observed mass | Calculated mass | Error (ppm) | Proposed neutral loss |
| --- | --- | --- | --- | --- | --- |
| Glycyrrhetic acid | C_30_H_47_O_4_ | 471.3488 | 471.3474 | 2.895 | - |
| (peak 3) | C_30_H_45_O_3_ | 453.3345 | 453.3368 | - 5.2295 | H_2_O |
|  | C_29_H_45_O_2_ | 425.3420 | 425.3419 | 0.1032 | HCOOH |
|  | C_29_H_43_O | 407.3313 | 407.3303 | - 0.2242 | H_2_O |
|  | C_28_H_39_ | 389.3194 | 389.3208 | - 3.6644 | C_14_H_16_ |
|  | C_20_H_29_O_3_ | 317.2127 | 317.2116 | 3.2467 | C_10_H_16_ |
|  | C_19_H_27_O | 271.2069 | 271.2061 | 2.6152 | HCOOH |
|  | C_16_H_23_O_3_ | 263.1663 | 263.1647 | 6.0044 | C_14_H_11_ |
|  | C_15_H_23_O_2_ | 235.1703 | 235.1698 | 2.104 | CO |
|  | C_15_H_21_O | 217.1600 | 217.1592 | 3.9975 | HCOOH |
|  | C_14_H_22_ | 189.1647 | 189.1643 | 1.9779 | HCOOH |
|  | C_10_H_13_O | 149.0971 | 149.0966 | 3.084 | C_5_H_9_ |
| Peak 4 | C_30_H_49_O_3_ | 457.3683 | 457.3681 | 0.2823 | - |
|  | C_30_H_47_O_2_ | 439.3574 | 439.3576 | - 0.4692 | H_2_O |
|  | C_29_H_47_O | 411.3608 | 411.3626 | - 4.5982 | HCOOH |
|  | C_29_H_45_ | 393.3516 | 393.3521 | - 1.3392 | H_2_O |
|  | C_16_H_24_O_2_ | 249.1876 | 249.1854 | 8.6068 | C_14_H_22_ |
|  | C_15_H_23_ | 203.1794 | 203.1799 | - 2.8345 | HCOOH |
|  | C_14_H_23_ | 191.1794 | 191.1799 | -3.0124 | C_16_H_24_ |
